# Childhood obesity, dietary patterns and gut microbiota: a narrative review

**DOI:** 10.1007/s13105-026-01142-w

**Published:** 2026-01-14

**Authors:** Natalia Vázquez-Bolea, Santiago Navas-Carretero, Marta Cuervo

**Affiliations:** 1 Department of Nutrition, Food Sciences and Physiology, Faculty of Pharmacy and Nutrition, University of Navarra, Pamplona, 31008 Spain; 2 Center for Nutrition Research, University of Navarra, Pamplona, 31008 Spain; 3 Biomedical Research Networking Center for Physiopathology of Obesity and Nutrition (CIBERObn), Institute of Health Carlos III, Madrid, 28029 Spain; 4 Navarra Institute for Health Research (IdiSNA), Pamplona, 31008 Spain

**Keywords:** Gut microbiota, Childhood obesity, Dietary patterns, Gut modulation

## Abstract

Childhood obesity is an emerging and multifactorial public health challenge resulting from the interaction of genetic, metabolic, social, and cultural determinants. Dietary habits are particularly relevant, as they are central to the regulation of energy balance. Indeed, gut microbiota seems to be a target factor to prevent and treat obesity. This narrative review aims to evaluate the gut microbiota in children suffering from obesity, and to assess how different dietary patterns affect these children’s gut microbiota. A comprehensive literature review conducted through the PubMed database identified 20 studies on gut microbiota and obesity in children, and 15 studies on the impact of different diets (Vegetarian, Western, Mediterranean) on the gut microbiota, with bacterial taxa names updated to the latest 2024 nomenclature. The *bacillota* phylum appears to be increased in individuals with obesity, while the *bacteroidota* phylum, on the contrary, is decreased, resulting in an increased *Bacillota/Bacteroidota* (B/B) ratio in children leading with obesity. The Mediterranean diet has been shown to improve bacterial diversity and to reduce inflammation, contrary to the Western diet (WD). Vegetarian diets have been shown to be a beneficial way to promote Short Chain Fatty Acids (SCFA) production. Additional larger studies are needed to evaluate this issue, as differences in findings are observed, especially in pediatric population, when assessing the gut microbiota.

## Key points

Children with obesity show an increased *bacillota* and a decreased *bacteroidota* phylum.

Pediatric subjects with obesity appear to have a higher *bacillota/bacteroidota* (B/B) ratio.

The Mediterranean and Vegetarian diets enhance bacteria diversity, contrary to the Western diet.

## Introduction

### Childhood obesity

Obesity rates have increased during the last decades, being considered as a crucial public health issue. According to the most recent data published from the World Health Organization (WHO), in 2022 one in eight people lived with obesity. Among children and adolescents between 5 and 19 years of age, more than 390 million suffered from overweight including 160 million living with obesity (1). In Spain, the ALADINO study in 2023 showed that more than 17% of Spanish children aged 6 to 9 suffered from obesity (2).

The WHO defines obesity as an abnormal or excessive fat accumulation [1]. In adults, obesity is defined as having a body mass index (BMI) over 30 kg/m^2^ [1]. However, in children, BMI reference is not useful for young people, as the age needs to be considered, thus growth curves were developed by the WHO. For children under 5 years old, obesity is defined as presenting a weight-for-height or a BMI for age over 3 standard deviations above the median Child Growth Standards, while for children between 5 and 19 years old, above 2 standard deviations from the WHO reference median [3]. The “Fundación Faustino Orbegozo” developed specific growth curves for children. Those above the 97th percentile, considering the BMI for age, were considered as suffering from obesity [4].

Experiencing obesity at a young age, may increase the risk of adult obesity by five times [5]. Indeed, more than half of children who develop obesity during childhood are likely to continue experiencing obesity into adolescence, and of those adolescents suffering from excess body weight, up to 80% will continue in the adulthood. This early onset of obesity will lead to various health complications, including cardiovascular problems like hypertension and atherosclerosis [6, 7], metabolic disorders such as dyslipidemia, insulin resistance, prediabetes or type 2 diabetes mellitus, non-alcoholic fatty liver disease [6, 7], gastrointestinal problems such as reflux [6], pulmonary conditions such as asthma and sleep apnea [6–8], as well as orthopedic complications like osteoarthritis [6].

Causes of obesity are wide ranged [9], and given this multifactorial etiology, many conditions play a role, such as genetics, biological, psychological, behavioral, environmental, individual, sociodemographic, health and socioeconomic status [10]. All these items could affect energy intake, which may result in a caloric imbalance due to consuming more calories than expended, leading to excess body weight [11]. Indeed, another factor that is under study and may play a key role in the development of obesity is the gut microbiota, as it has been lately considered as an endocrine organ with the ability of maintaining energy homeostasis [12], body weight control and inflammation, thus playing a potential role in the onset of obesity [13]. Notably, the gut microbiota has been found to have a relevant impact on overall energy balance, as it is involved in the entire digestion process through interactions between microorganisms and the host´s metabolism [14], so it can be a potential target for treating and preventing obesity [15]. In addition, different dietary patterns seem to alter the composition of the gut microbiota, and thus, have a crucial impact on weight and adiposity [16], as well as in the adequate functioning of the organism, as the interaction with the host leads to the synthesis of metabolites that exert either positive or negative effects on human health [17].

Although gut microbiota undergoes changes along the different stages of life [18], it has fundamental roles in immunological, metabolic, structural and neurological aspects of the human body. In addition, it also plays a role in the physical and mental well-being of individuals [19]. Indeed, gut microbiota not only acts on the digestive tract, but also sends and receives signals to and from the brain which can regulate appetite and act on eating behavior. This is commonly known as the “gut-brain axis”, a bidirectional connection between the gut and brain through the central and the enteric nervous system, mainly by neural, endocrine and immunological pathways [20, 21].

### Gut microbiota. Formation, composition and functions

The gut microbiota refers to the complex and diverse community of trillions of microorganisms that reside in the gastrointestinal tract, which include predominantly bacteria, but also viruses, and single-cells eukaryotes as protozoa and fungi [22, 23]. With regards to bacteria, more than 1500 different species exist, with different composition/richness in each part of the gastrointestinal tract, varying in the stomach, small and large intestine [24, 25]. Gut microbiota is composed of numerous bacteria species classified by genus, family, order, class and phylum [26]. Metagenomic studies refer that 90% of the total microbiota is constituted by five different phyla: *bacteroidota*,* bacillota*,* actinomycetota*,* pseudomonadota*,* and verrucomicrobiota*, although more than 50 phyla shape the gut microbiota, but the first two are the most prevalent ones [25]. First, *bacteroidota* stand out the most, both by being one of the most abundant species, up to 30% of the total microbiota, and by participating in numerous physiological processes for optimal body functioning [27]. *Bacteroidota* phylum, which is gram-negative, is made up by *bacteroides* and *prevotella* genera [28]. Going to the next phylum in importance, *bacillota* is gram-positive and is composed mainly by *lactobacillus*,* bacillus*,* clostridium*,* enterococcus and ruminococcus* genera [26]. In addition, regarding *actinomycetota* phylum, *bifidobacterium* is the most common genus in it [29]. However, it is important to note that up to date, there is no healthy microbiota composition, as it varies between individuals [26]. When studying gut microbiota, we can differentiate between α-diversity, which measures the richness and abundance of microbiota species within one sample; and β-diversity which measures the variability within two different communities, to look for similarities or dissimilarities [30].

To identify the composition of the microbiota, three different methods can be used [31, 32]. The first is bacterial culture, which allows the quantification of viable microorganisms; however, it presents a major limitation, as only around 20% of bacterial species are culturable under standard laboratory conditions [33]. The second approach is metataxonomics, defined as the estimation of the total DNA taxonomic composition through next-generation sequencing of the 16 S rRNA gene. This widely used technique targets conserved and variable regions of the 16 S rRNA gene to enable community-wide taxonomic classification. Nevertheless, it requires PCR amplification, which may cause several methodological limitations and biases. Primer mismatches can result in preferential amplification of certain taxa, as primers bind to regions that are not fully conserved across all bacterial species, leading to distorted estimates of community composition [34]. Moreover, because this method targets only a small region of the bacterial genome, its taxonomic resolution is limited, typically at the genus level, and it cannot reliably discriminate between closely related species. The third approach, metagenomics, overcomes PCR-related biases and is not restricted solely to bacterial sequences. By covering the entire genomic content of the sample beyond the 16 S rRNA gene, shotgun metagenomic sequencing enables comprehensive taxonomic identification and functional characterization across all microbial domains.

The formation of the gut microbiota starts as early as during gestation. Studies have shown that the mothers’ placenta contains these microorganisms, indicating that transmissions between mother and child can occur during this period [35]. The children’s gut microbiota formation is a dynamic process, affected by numerous factors. The mode of delivery influences it, observing more diversity through vaginal delivery than with cesarean one [36]. The type of feeding, more precisely the introduction of complementary feeding or the children´s diet can also modulate the microbiota [37], as well as the consumption of antibiotics, which reduces the diversity of it [36]. Indeed, the microbiota is not firmly established until the age of three, when it can then be compared to the microbiota of adults [38]. This period is essential, since the microbiota of children responds much more to external factors and environmental influences than the one in adults [39]. The age [40], as well as the environment, gender, location, genetics, pets, maternal diet, climate, habits, exercise or BMI are other factors that can affect the composition of the gut microbiota [26, 41]. However, although several factors seem to modulate the microbiota, the diet appears to be the most relevant one, highlighting that the study of it is of great importance for thoroughly understanding the physiological state of children [41].

### Gut microbiota and obesity: biological mechanisms

Gut microbiota is thought to play a key role in the development of obesity, as it is involved in energy homeostasis, participating in extracting energy by fermentation and formation of SCFA [42]. Increased concentrations of SCFA, including propionate, acetate and butyrate have been observed in children with obesity. Studies report that the *bacillota* phylum is the most effective in extracting energy from the bolus that reach the large intestine, which increases the amount of energy absorbed and therefore the probability of gaining weight [42]. This fact may explain why children with obesity appear to have increased concentrations of *bacillota* phylum, and a decrease in the *bacteroidota* phylum. In that sense, the *B/B* ratio in children is of interest and differences in this ratio have been observed when comparing children that present obesity or healthy ones [43]. Indeed, a situation that can be found in the gut microbiota is dysbiosis. This occurs when there is a decrease in beneficial species and an expansion of non-beneficial species, leading to both a loss of diversity and an alteration in the composition and functional activity of the gut microbiota compared to that observed in healthy individuals [44]. In this context, the aim of this narrative review was to examine the microbiota of children with obesity, with a particular focus on the *bacillota/bacteroidota* ratio, specific microbiota taxa and the relationship between childhood obesity and SCFA. In addition, a secondary objective was to differentiate the gut composition depending on their dietary patterns, regardless of weight status.

## Methodology

A comprehensive literature search was conducted using the PubMed database to identify relevant studies. To ensure the relevance and quality of the selected studies, only articles published in English before May 2024 were included. Studies analyzing gut microbiota in individuals aged 0 to 18 years were selected. The variability in participant age ranges across individual studies is identified as a methodological limitation of this review, which will be addressed in the discussion. For the first objective, we employed a combination of the following keywords: *Gut microbiota*,* child*,* obesity*,* firmicutes*,* bacteroidetes and firmicutes/bacteroidetes ratio*. After an extensive and thorough review of the literature, a total of 20 original articles were selected that met the criteria established for this review. Additionally, we further searched for the role of SCFA on specific articles derived from those originally chosen. For the second objective, we searched for studies using the keywords *Vegetarian diet*,* Western diet*,* Mediterranean diet*,* child*, and g*ut microbiota*. Following a review of the literature, a total of 15 studies were selected based on their relevance to the research question. After careful assessment, we ultimately retained four studies focused on the Vegetarian diet (VD), five studies examining the Western diet (WD), and six studies that investigated the Mediterranean diet (MD). In this review, the names of the bacteria taxa have been updated according to the latest 2024 nomenclature [45], to reflect the most recent taxonomic updates and to promote more inclusive and precise scientific communication. However, it is important to note that the original articles included in this review used previous nomenclature.

## *Bacillota*/*Bacteroidota* (B/B) in childhood obesity

An increase in the ratio *B/B* is a condition that appears to be common in population suffering from obesity. Although there are numerous bacterial species, alterations in the *bacillota* and *bacteroidota* phyla seem predominant when suffering from obesity. Indeed, one of the first studies performed in 2005 in mice already showed these findings: the *B/B* ratio was increased in obese mice and decreased in lean ones [46]. One of the latest systematic reviews performed in 2020 included numerous studies in humans and showed the same results: individuals with obesity presented a higher *B/B* ratio [47]. Thus, *B/B* ratio seems to be an indicator of obesity in adults. However, in children, few articles have been published about *B/B* ratio; little is known, and results appear to be contradictory (Table 1).

**Table 1:**
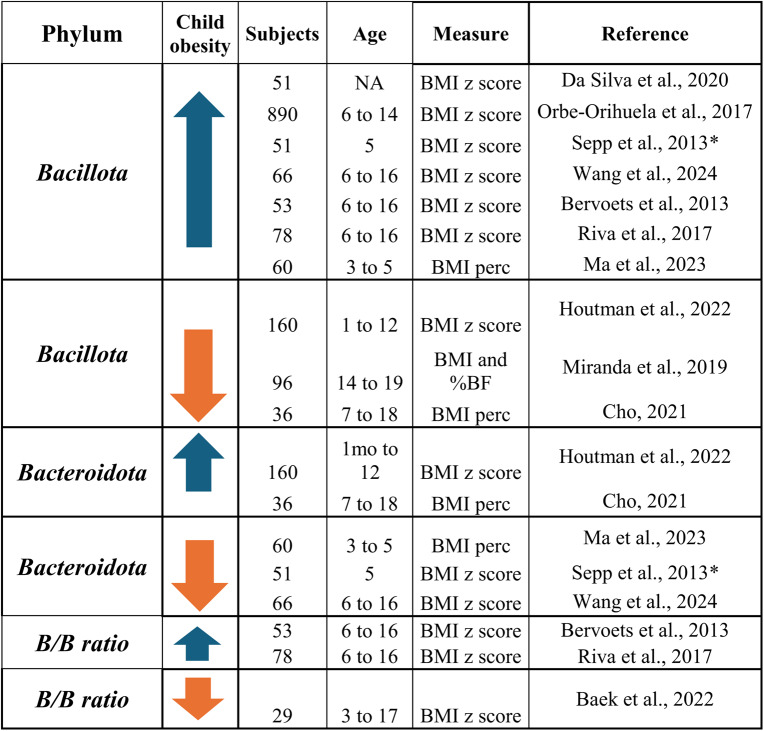
*Bacillota, bacteroidota*and*B/B* ratio in childhood obesity

* Studies in which cultures have been used.

Abreviations: B/B ratio= Bacillota/Bacteroidota ratio; BMI=body mass index; BF=body fat; perc=percentile

If we compare the *bacillota* phylum in children with obesity against healthy weight ones, the abundance of this phylum seems to increase along with the BMI [48, 49]. A recent study in southeastern China in 2024 reported that the proportion of *bacillota* considerably increased from 49.8% in normal-weight children to 63.5% in children suffering from obesity, whereas the proportion of *bacteroidota* decreased from 37.7% to 18.5% in children living with obesity [50]. Other numerous studies support these same findings [51, 52]. Indiani et al. indicated a decrease in *bacteroidota* phylum and in *bacteroides and prevotella* genera among children with higher BMI, as well as an increase in *bacillota* phylum associated to a higher abundance of *clostridium*,* eubacterium* and *lactobacillus* [52]. Ma et al. reported an increase in *bacillota* and a decrease in *bacteroidota* phylum in the intestinal microbiota of children presenting obesity, highlighting some genus as *faecalibacterium*, *tyzzerella* or *klebsiella*, which could be considered as biomarkers for obesity in children aged 3 to 5 years [53]. Another study in older children found that within the phylum *bacillota*, the species *streptococcus thermophilus* and the genus *acidaminococcus*; within the phylum *bacteroidota*, the species *prevotella copri;* and within the phylum *pseudomonadota*, the species *sutterella wadsworthensis* were all positively associated with obesity [54]. Thus, an increase in *bacillota* phylum and a decrease in the *bacteroidota* one entails an increase in *B/B* ratio, as observed in various studies performed in children [55, 56].

However, not all the bibliography supports these findings. Several studies found no relationship between the *B/B* ratio and BMI, even finding a lower *B/B* ratio in children with obesity. Houtman et al. aimed to explore whether the *bacillota* phylum or the *bacteroidota* one was each individually associated to obesity during infancy and found no correlation to it [57]. Indeed, a study in Korean children found no significant difference between *B/B* ratio in children with obesity and those with normal weight [60]. Another study performed only in girls found a positive association between *bacillota* phylum and waist circumference, but not with BMI or body fat percentage [58]. Cho et al. performed a nutritional intervention in children aged 7 to 18. Children who achieved a reduction in body fat presented a decrease in *bacteroidota* phylum (*bacteroides genus*), and an increase in the *bacillota* phylum (*clostridia genus*), whereas participants who increased weight and body fat, presented a decrease on the *bacillota* phylum (*clostridia genus*), contrary to what was expected [59].

In this sense, although it is common to use the *B/B* ratio to evaluate the association of gut microbiota with ponderal status, heterogeneity is common when assessing it in children. Indeed, recent findings support the idea that instead of using the *B/B* ratio for associating the presence of obesity, SCFA production could be of interest [56]. The results obtained from the studies included in this review are highly heterogeneous, which may be explained by several factors. First, the populations analyzed differ in terms of age, developmental stage, and BMI, all of which are known to influence gut microbiota composition. Moreover, lifestyle-related factors such as diet, physical activity, and environmental exposures may also contribute to these discrepancies. Methodological aspects play a crucial role as well, since the studies employed different approaches for microbiota characterization each with distinct taxonomic resolution. In addition, the observed variability might also be related to differences in gender, genetic background, and environmental conditions, as the target population is heterogeneous and undergoing a growth period that involves significant physiological changes affecting gut microbiota stability and composition. Given the limited number of studies addressing this topic in children, further research with larger sample sizes and standardized methodologies is needed to confirm these findings. Therefore, the B/B ratio is not a reliable biomarker for childhood obesity. Given these limitations, examining microbial metabolites such as SCFAs may offer a more precise and mechanistic understanding of how gut microbiota influences pediatric obesity.

Beyond microbiota compositional analyses, the functional effects of the gut microbiota, particularly SCFAs, provide critical insights into the mechanisms that link the microbiota and pediatric obesity. The quantity and quality of dietary fiber strongly influence gut composition and, consequently, SCFA production. SCFAs (mainly acetate, propionate, and butyrate) are essential for metabolic health, contributing up to 10% of daily caloric requirements and serving as the main energy substrate for colonocytes, while also supporting intestinal barrier function by reducing permeability [61]. Through interaction with G protein receptors (GPR41 and GPR43) [62], they modulate enteroendocrine hormones such as GLP-1 and PYY [63], thereby influencing appetite regulation, lipogenesis, glucose homeostasis, and overall energy balance [61, 64]. Early life SCFA exposure, affected by maternal diet, delivery mode or breastmilk composition, may differentially program immune responses and adiposity trajectories in children [65–68]. Additionally, SCFAs act as critical signaling molecules in the gut-brain axis, influencing neurodevelopment, neuroinflammation, and behavior, with implications for pediatric obesity, anxiety, depression, and neurodevelopmental disorders [69]. Across reviewed studies, SCFAs consistently appear as central mediators between gut microbiota composition and obesity in children, but their role is complex and context-dependent. Some studies reported reduced butyrate and isobutyrate and increased caproate concentrations in children with obesity, reflecting disrupted fermentation pathways [70]. These changes were accompanied by a decrease in the abundance of butyrate producing bacteria such as *Oscillibacter* and increased activity of potentially harmful fecal enzymes (β-glucosidase, β-glucuronidase). This supports the idea that obesity-related microbial shifts impair the production of beneficial metabolites and promote gut-derived metabolic stress [71]. In contrast, other studies found elevated fecal SCFA concentrations in children with obesity (notably acetate, propionate, and butyrate) [72], as well as positive correlations between total SCFAs and regional body fat distribution, including android and gynoid regions [73]. These results indicate that elevated SCFA levels may not always reflect protective metabolic activity but could instead represent increased microbial fermentation due to greater substrate availability and caloric intake. Indeed, recent evidence suggests that alterations in SCFA levels observed in obesity may result from gut microbiota dysbiosis, leading to disrupted fermentation processes and metabolic imbalances [74]. Other studies highlight the dual role of SCFAs. On one hand, they serve as an energy source for colonocytes, enhance intestinal barrier integrity, and modulate appetite-regulating hormones such as GLP-1 and PYY through G protein-coupled receptors. On the other hand, excessive SCFA production may contribute additional calories, influence insulin secretion, and promote lipid storage, thereby favoring weight gain under certain metabolic contexts [64]. Overall, the evidence highlights a dual role of SCFAs in pediatric obesity. While adequate SCFA production supports intestinal and metabolic health, excessive or dysregulated SCFA generation may provide additional energy to the host and promote adiposity. Thus, SCFA metabolism may act both as a marker and mediator of obesity-related microbial alterations. The direction of this association, protective or pathogenic, appears to depend on microbial composition, substrate availability, and host metabolic context. Future longitudinal and mechanistic studies are needed to determine whether targeted modulation of SCFA production through dietary or microbiota-based interventions could effectively prevent or manage childhood obesity.

## Dietary patterns and gut microbiota

As discussed previously, diet plays a key role in the development, maturation, and modulation of gut microbiota in children. A detailed understanding of how different dietary patterns influence pediatric gut microbiota is still needed. The composition of the diet, the proportion of macro and micronutrients and their sources will thus play a key role in shaping the gut microbiota. Dietary changes can modulate its composition rapidly and thus, affect the host´s health and metabolism [75]. Establishing enduring healthy dietary patterns is vital not only for influencing human health but also for maintaining a rich diversity and abundant microbial ecosystem within the gastrointestinal tract, commonly referred to as “eubiosis,” while preventing the appearance of “dysbiosis” [76].

It is important to note that breastfeeding or milk formulas have a significant impact on the gut composition. Breastfeeding has been associated with increased abundance of beneficial *bifidobacterium*, improved immune system function, reduced risk of infections, and a more stable gut microbiota [77]. One of the last metanalysis concluded that breastfeeding is essential for the homeostatic development of the infant gut microbiota, highlighting the short- and long-term benefits that entails it [78]. All this is probably due to the presence of human milk oligosaccharides (galactooligosaccharides) which are the third most abundant components and are present in maternal milk [79]. They play a key role for inhibiting the growth of pathogens’ families such as *clostridiaceae*,* enterobacteriaceae*, and *staphylococcaceae* through the production of SCFA [80]. On the other hand, formula feeding has been positively associated with increased microbiota diversity, particularly in *clostridiales* and *pseudomonadales*, but with lower levels of *bifidobacteriales* order. It has also been correlated with a more diverse and mature microbiota, and a gut composition like the adult one [78]. In addition, it has been associated with increased bacterial pathogens, which could have an impact on human health [77].

The introduction of complementary feeding plays an important role in shaping the gut microbiota. After breastfeeding or formula-feeding, the transition to the introduction of solid foods is linked to a change in both the structural and functional microbiota diversity of the infant, whose final goal is the establishment of a mature intestinal microbiota similar to the one of adults [76]. Substantial changes tend to occur in this period, characterized by an increase in diversity, accompanied by alterations in communities of bacteria [81]. This transition involves a shift from a predominance of *bifidobacterium* (*actinomycetota* phylum), to communities dominated by *bacteroidota* and *bacillota* [82]. The introduction of solid foods fosters the growth of beneficial taxa such as *bifidobacterium*,* lactobacillus*, and *bacteroides* [83]. Vegetable consumption, rich in fermentable fibers, enhances the populations of *bacteroides* and *bifidobacterium* [84]; while protein consumption increases levels of *clostridium* and *streptococcus* bacteria [85]. Thus, considerable changes tend to happen in microbiota composition. The timing of introducing solid foods is also of relevant interest [82]. Early introduction of complementary feeding has been linked to an increased exposure to allergens, bacterial pathogens as well as possible alterations in the immune system and oxidative stress [86, 87]. Research indicates that introducing complementary feeding before 3 months of age may lead to alterations in gut microbiota composition and SCFA profiles. Although butyrate is generally considered beneficial due to its anti-inflammatory and gut barrier protective properties, early shifts in gut microbiota may indicate imbalances in SCFA production or microbial function, potentially influencing metabolic outcomes and increasing susceptibility to obesity later in life [82]. Conversely, a delayed introduction of complementary feeding has been associated with slower maturation and development of the gut microbiota [87]. Therefore, achieving an optimal timing is essential, with current recommendations from the European Society for Paediatric Gastroenterology, Hepatology and Nutrition (ESPGHAN) suggesting the introduction of complementary foods no later than 6 months of age to ensure the greatest health benefits [88].

**Fig. 1:**
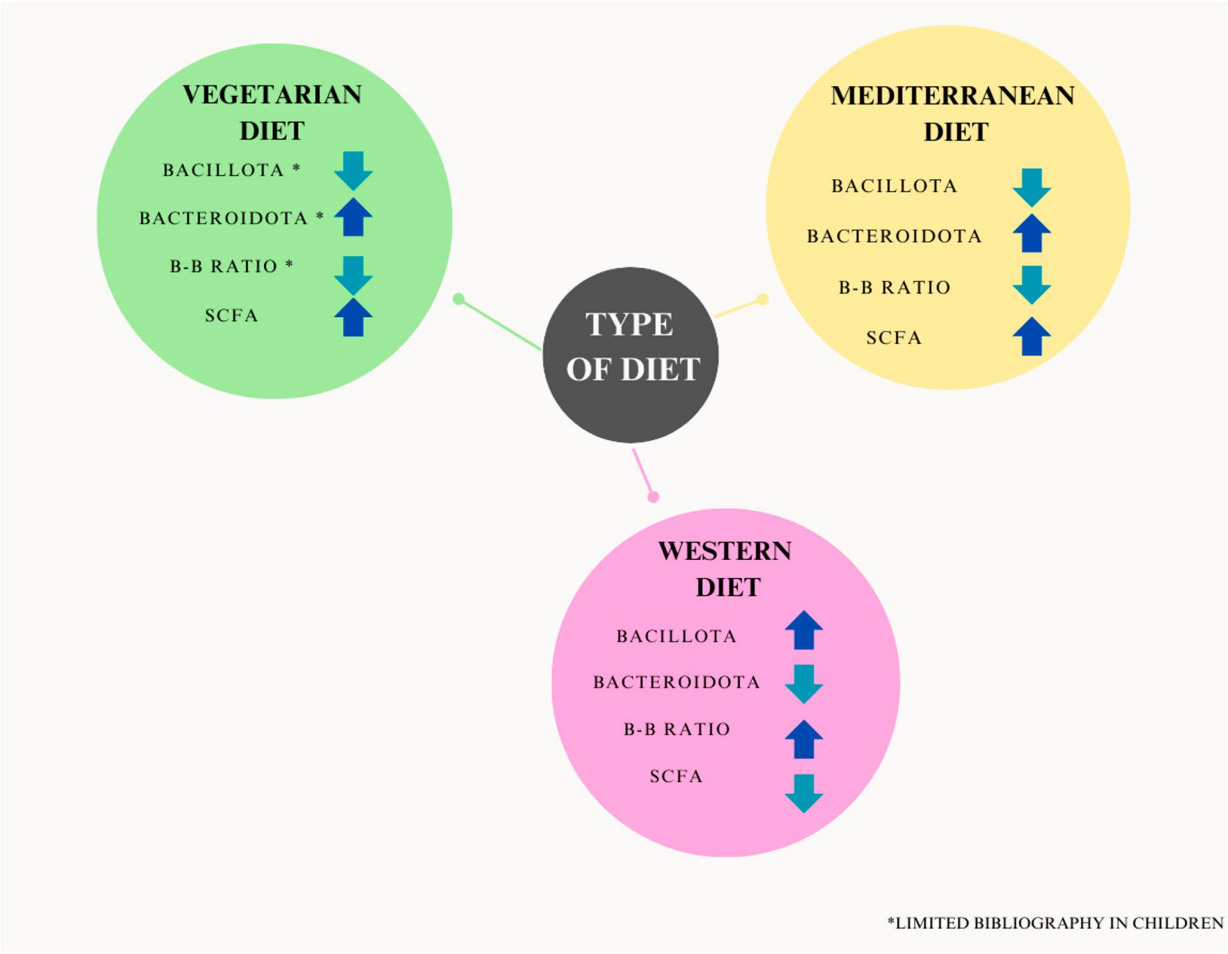
Diets, *bacillota* and *bacteroidota* phylum, *B/B* ratio, and SCFA production

After the introduction of complementary feeding, children may adopt various dietary patterns influenced by factors such as family preferences, cultural habits, socioeconomic status, geographic location, and other variables that impact nutritional habits. In that sense, our dietary choices play a significant role in shaping the gut microbiota, with different dietary patterns exerting distinct effects on microbiota composition (Fig. 1; Table 2).

**Table 2:**
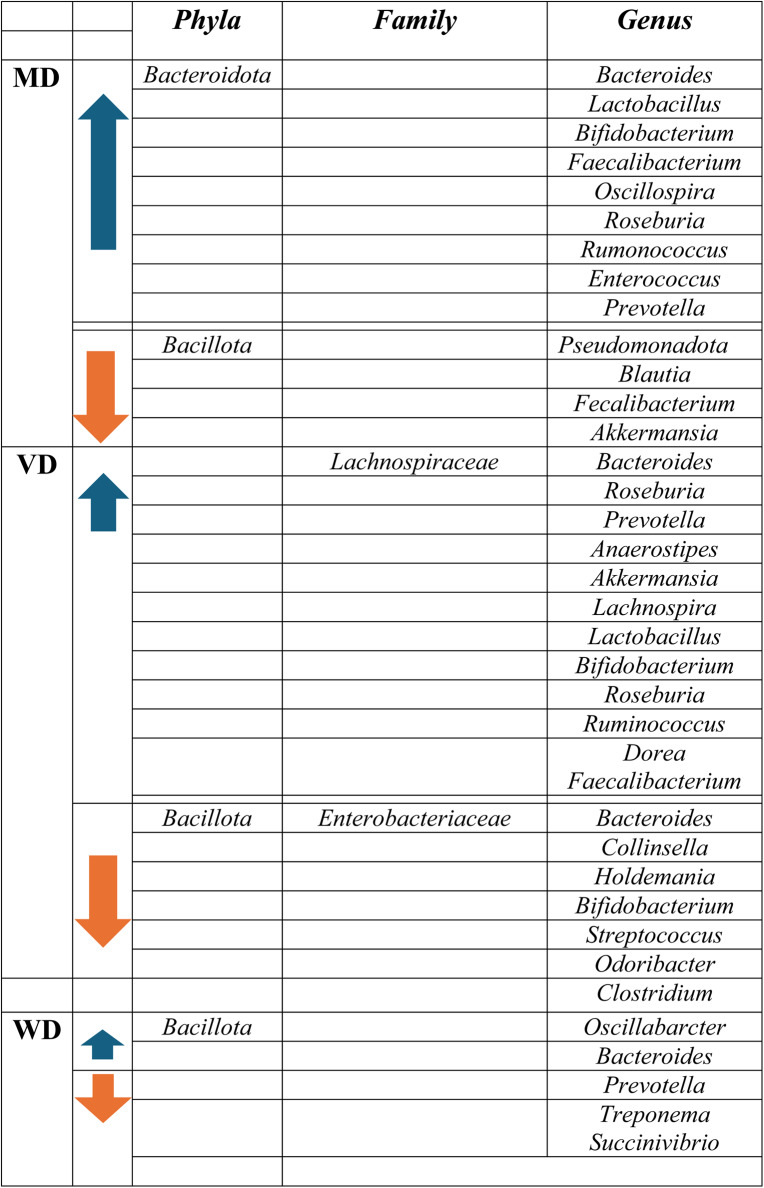
Diets and microbiota composition associated to them

*MD*=Mediterranean Diet; *VD*=Vegan Diet; *WD*=Western Diet.

In this context, we opted for examining the Western, Mediterranean and Vegetarian diets. These dietary patterns were selected because they represent distinct dietary approaches with substantial differences in food composition and nutrient intake. The Western diet is typically high in processed foods, saturated fats, and refined sugars, which have been associated with alterations in gut microbiota composition. In contrast, the Mediterranean diet is rich in fiber, healthy fats, and polyphenols, which are known to promote microbial diversity and beneficial gut bacteria. Meanwhile, the vegetarian diet is primarily plant-based, leading to a higher intake of fiber and plant-derived bioactive compounds, which can significantly influence gut microbiota composition. By examining these three contrasting dietary patterns, we aim to better understand their potential impact on the gut microbiota of children, regardless of weight status.

### Mediterranean dietary pattern

The Mediterranean Diet (MD) can be defined as a healthy dietary pattern prevalent in Mediterranean countries as Spain, Greece or Italy whose intake is primarily based on cereals, legumes, vegetables, fruits and olive oil [89]. It has countless health benefits, many of them associated to the type of fat present (olive oil, fish, and nuts), to the proportion of macronutrients (carbohydrates = 40–65%; fat = 28–40%; protein = 10–35%) [90] and to the abundance of micronutrients, derived from the consumption of vegetables, aromatic herbs, and spices. The decalogue of the MD encompasses the following statements: elevated consumption of extra virgin olive oil, vegetable origin products, particularly greens, fruits, cereals, nuts, and legumes, with moderated intake of fish, meat, dairy products, and red wine. Additionally, they advocate for a moderate consumption of eggs and a limited and occasional consumption of sweets [91].

In that sense, when evaluating the effect of the MD on gut microbiota, most of the studies were performed in adults. The MD has been associated with greater bacterial diversity, enriching the gut microbiota [92] and being favorable for overall health [93]. Data demonstrate that the microbiota from individuals who tend to adopt a Mediterranean dietary pattern differs drastically from the ones following a western pattern [93], and the MD has even shown effects in reducing dysbiosis [94]. Regarding taxa, the MD has been associated to an increase in *bacteroidota* phylum. Studies indicate that this may be attributed to the consumption of fiber, present in carbohydrates and vegetables [95], and protein [75]. Indeed, a decrease in *bacillota* phylum is also typical when following a MD pattern, which all this makes the MD to be associated with a lower *B/B* ratio [96]. In adults, diets rich in fiber and unsaturated fatty acids, as MD, are associated with high levels of beneficial bacteria that contribute to the reduction of inflammation, oxidative stress and overall health [97], such as *bacteroides*,* lactobacilli*,* bifidobacteria*,* faecalibacterium*,* oscillospira*,* roseburia*,* ruminococci*,* clostridium cluster*,* ruminiclostridium leptum*,* eubacterium rectale*; and lower levels of *bacillota*,* pseudomonadota and blautia* [93, 97, 98]. Another study found the MD significantly increased the abundance of *enterorhabdus*,* lachnoclostridium* and *parabacteroides*, as well as increasing the SCFA production, contributing to the protective properties and lower inflammation associated to the MD [99]. One last study mentioned that polyphenols associated to the dietary compounds (fruits, vegetables, cereals, tea, coffee) of the MD increased the number of *enterococcus*,* prevotella*,* bacteroides*, and *bifidobacterium* [98].

The literature about the impact of diets on microbiota composition in children is still limited. A comparative study was performed to examine microbiota disparities between children and adolescents in Egypt and in the U.S., who adhered to MD and WD, respectively. The findings indicated that Egyptian individuals exhibited a microbiota predominantly composed of *prevotella*, whereas the U.S. samples were dominated by *bacteroides*. Children from Egypt showed higher levels of SCFA and tended to experience greater bacteriophage pressure. Conversely, the gut of U.S. children exhibited greater richness in amino acids and lipid metabolism-related compounds; had greater number of microbial genes that encode protein degradation, vitamin biosynthesis, and iron acquisition pathways; and was enriched with protein and starch degrading genera [100]. Interestingly, the abundances of f*aecalibacterium* and *akkermansia*, two genera recognized for their anti-inflammatory and gut barrier–protective properties, were elevated in the guts of U.S. individuals [100]. This result may appear counterintuitive, as a Western dietary pattern is typically associated with a reduction in a*kkermansia muciniphila* richness, a key mucin-degrading bacterium crucial for maintaining intestinal integrity [101]. However, several factors could explain these findings. First, the Western diet is highly heterogeneous across populations, and its composition may differ substantially depending on fat quality, fiber, and polyphenol intake, which are known to modulate *akkermansia* and *faecalibacterium* abundance. For instance, higher consumption of unsaturated fats or polyphenol-rich foods within certain subgroups may counterbalance the detrimental effects of saturated fat intake. Second, *akkermansia* abundance is strongly influenced by host-related factors such as metabolic status or antibiotic exposure, which may vary between cohorts and partially explain interpopulation differences [102, 103]. Furthermore, genus-level taxonomic resolution derived from 16 S rRNA sequencing might mask species or strain specific responses, as not all *akkermansia* lineages exhibit the same ecological or metabolic traits. Therefore, the observed enrichment of these beneficial taxa in the U.S. group likely reflects a complex interaction between dietary composition, lifestyle, and host physiology rather than a direct consequence of the Western diet.

### Vegetarian and flexitarian dietary patterns

According to the Spanish Paediatric Association, vegetarian diets are “those that are free of meat and meat products and fish (including shellfish and its derivatives)”. According to the type of vegetarian diet, “it can include eggs or dairy products (ovo/lacto-vegetarian) or exclude any products of animal origin, including honey (vegan diet)” [104]. In addition, a flexitarian diet includes all the aforementioned characteristics, but it can include meat consumption in reduced amounts [105]. These dietary patterns, such as the Mediterranean and vegetarian diets, have been associated with a reduced risk of obesity and other non-communicable diseases. However, certain vegan diets may require specific nutrient supplementation, particularly vitamin B12, to ensure adequate nutritional intake and prevent potential deficiencies [106].

A study conducted in children from a rural area of Burkina Faso (BF) and Europe revealed that kids from BF exhibited a higher proportion of *bacteroidota* phylum and a lower one of *bacillota* phylum (73% *bacteroidota* and 12% *bacillota*), compared to children from different areas of Europe (27% *bacteroidota* and 51% *bacillota*) [107]. These differences are likely related to dietary patterns, as children from BF consume diets rich in complex carbohydrates derived from plant-based foods, whereas European diets are increasingly characterized by higher saturated fat intake and lower fiber content due to the global adoption of Westernized eating habits. Although the traditional Mediterranean diet, originating in Europe, is rich in fats, these are predominantly unsaturated and associated with beneficial effects on gut microbiota composition. Consumption of plant-based products containing dietary fibers serves as the primary energy source for gut-residing microorganisms, involved in the production of acetate, propionate, and butyrate [108]. The microbiota species associated with elevated SCFA production include a*kkermansia*,* lachnospira*,* lactobacillus*,* bifidobacterium*,* roseburia*,* ruminococcus*,* clostridium*,* faecalibacterium*,* and dorea* [109]. *Akkermansia*, specially *akkermansia muciniphila*, plays a key role in maintaining the intestinal barrier by degrading mucin and has been linked to improved metabolic profiles and reduced inflammation [110]. *Lactobacillus* and *bifidobacterium* are well-known beneficial genera involved in carbohydrate fermentation, lactic acid production, immune modulation, and protection against pathogens [111, 112]. *Lachnospira*,* dorea*,* roseburia*,* faecalibacterium*,* ruminococcus*, and certain *clostridium* are important SCFAs producers, mainly butyrate, contributing to colonic epithelial health and exhibiting anti-inflammatory properties. Altogether, these taxa contribute to gut homeostasis, metabolic regulation, and host immune balance. Another study found that vegan diets changed the abundance of the following bacteria genera, increasing *anaerostipes* and decreasing *streptococcus*,* clostridium sensustricto*, and *odoribacter* [99]. This may be beneficial for children, as a higher abundance of *anaerostipes* has been linked to lower bone age and reduced levels of hormones such as LH and FSH [113]. However, a review performed by Glick-Bauer et al. found that vegan diets are related to lower levels of *bacteroides*,* bifidobacterium a*nd *enterobacteriaceae* [114], whereas Matijašić et al. referred that a vegetarian diet was associated with higher proportion of *bacteroides*,* prevotella*,* clostridium*, and *faecalibacterium* [115]. These differences can be associated to the wide variety of vegetarian diets, as the largest variation in microbiota composition is explained by the consumption of foods of animal origin (eggs, white meat, milk, yogurt, other dairy products, fish, and seafood), and thus, several vegetarian diets include and other exclude these dietary compounds [115]. From a physiological perspective, these bacterial shifts may have important implications for host metabolism and intestinal health. *Bacteroides* can produce all SCFAs, whereas *prevotella* is able to metabolize a wide variety of proteins and polysaccharides, producing propionate as one of its key fermentation metabolites [116, 117]. Its higher abundance in vegan diets is consistent with the increased fiber intake typical of such diets. *Clostridium* and *faecalibacterium* are major butyrate producers with anti-inflammatory properties that support colonocyte energy supply and intestinal homeostasis [117]. Pathogenic bacteria, especially members of the *enterobacteriaceae* family, and the toxic compounds they produce are associated with increased oxidative stress and inflammatory responses [118]. The reduction of these taxa in vegan diets contributes to lower inflammation and improved metabolic health. Children following vegan diets often show reduced levels of *bifidobacterium*, likely due to the absence of dairy products, which are a primary dietary source of these beneficial bacteria. This reduction may predispose to dysbiosis, affecting SCFA production and gut homeostasis [119]. Supplementation with *bifidobacterium* containing probiotics or fortified foods could help mitigate these effects. Consequently, the balance among these microbial groups may influence the overall metabolic and immunological effects of different plant based dietary patterns. It is also interesting to note that the microbiota of children whose mother consumed a vegetarian diet differed slightly. No differences were found in alpha diversity, but beta diversity was reduced in vegetarians. In addition, differences were seen in the abundance of several genera, specifically a reduction in *collinsella* and *holdemania*, and increases in the relative abundances of *roseburia* and *lachnospiraceae family* [120]. An increase in *roseburia* genus is particularly beneficial for children, as it has been associated with improvements in language, behavior, and motor cognitive skills [121]. In addition, increased abundance of *collinsella* has been linked to several conditions, including non-alcoholic steatohepatitis, pro-inflammatory dysbiosis, and type 2 diabetes [122]. The lower proportion of this bacterium in vegan diets may therefore contribute to their beneficial metabolic and anti-inflammatory effects. Definitively, available literature articles state that a vegetarian or vegan diet is beneficial to promote the ecosystem and to support the microbiota [123], although significant bacterial differences can be seen depending on the type of vegetarian diet consumed.

### Western dietary pattern

A Western Diet is characterized by an elevated consumption of fats, predominantly saturated fatty acids (SFA) and trans fatty acids (TFA), refined carbohydrates, sugar and salt. It includes palatable products such as fast food, soft drinks, highly processed foods, pre-packaged foods, red meat, alcohol, candy, sweets, fried foods, which are all high in unhealthy fats, sugar and salt, although there is no specific definition for it. This type of diet has been associated with obesity, type 2 diabetes and increased cardiovascular risk [124]. Gut microbiota also suffers alterations when consuming these foods. Available literature regarding WD states that the ratio of *B/B* increases when high-fat diets are consumed. This is associated to higher *bacillota* and lower *bacteroidota* phylum [125]. An increase in *B/B* ratio when consuming sugary products has also been observed. *Lactobacilus* and *bifidobacterium* also appear to decrease when consuming high fats diets, sugar, salt and additives [101, 126, 127]. A decrease richness of *akkermansia muciniphila* is also a common characteristic of a WD, which is an important bacteria for the maintenance of the gut barrier [101]. A reduced intake of dietary fiber, characteristic of the WD, limits the production of SCFAs, key microbial metabolites with recognized benefits for host metabolism, insulin sensitivity, and weight regulation, as mentioned. The consequent decrease in SCFAs can contribute to gut dysbiosis and impair intestinal barrier integrity, immune regulation, and overall homeostasis, potentially increasing inflammation and susceptibility to metabolic and immune-related disorders [109, 128, 129].

In children, less bibliography is available. However, some studies have assessed the impact of the Westernized dietary pattern on the microbiota. A study conducted in Leyte Island [130] suggests that increased fat consumption correlates positively with the *B/B* ratio and negatively with the prevalence of the *prevotella* family. At the species level, fat intake shows a positive association with certain *oscillibacter* and *bacteroides* species. *Oscillibacter* appears to be more prevalent in children with obesity, which may be related to higher intake of a Western diet and the development of obesity [70]. Notably, overweight and obese children with high-fat diets appear to have a microbiota with elevated *B/B* ratios and reduced *prevotella* abundance. These changes in gut microbiota composition, specially *prevotella*, could serve as indicators of obesity, as several articles have shown correlations for it [54, 131, 132]. Another study analyzed the microbiota in children from a rural area from BF compared to children from Florence (Italy) who consumed a WD [133]. It is interesting to note that African children had several unique bacterial associated to rural areas, were westernized dietary patterns are lacking. These include *prevotella*,* treponema*, and *succinivibrio*, species that are responsible for fermenting fibers from vegetables and polysaccharides, and are lacking when consuming a WD. *Prevotella*, which ferments polysaccharides and proteins to produce propionate, tends to be less abundant under low-fiber conditions [116], potentially reducing its beneficial effects on glucose regulation and satiety. In addition, the same study mentions that the proportion of SCFA was reduced in Florence (WD) in respect to BF, indicating the urbanization and WD are of interest for shaping and modifying the microbiota [133]. Indeed, children with undernutrition, which is a global health issue in non-developed countries from Sub-Saharan Africa and Asia, have increased *pseudomonadota* and decreased *bacteroides* [134]. This is consistent with other evidence indicating that *pseudomonadota* is more prevalent in stunted children, where it may contribute to disease, dysbiosis, and increased inflammation [135]. Another study that associated the dietary habits of American children with the microbiota showed that industrialized populations that consume food products high in animal protein and fats (WD) seem to have a lower diversity in the gut community [136].

This narrative review has some limitations that should be acknowledged. First, the inclusion of studies covering a broad pediatric age range (0–18 years) may introduce heterogeneity, as gut microbiota composition undergoes major developmental changes during the first years of life. Additionally, differences in study design, analytical techniques, and reporting methods further limit the comparability of findings across studies. Despite these limitations, this review provides a comprehensive overview of the current evidence linking gut microbiota and obesity in childhood.

## Conclusion

*B/B* ratio tends to be increased in children with obesity, although research is not consistent in this population. *Bacillota* phylum seems to be increased in children with excess weight, and *bacteroidota* phylum, on the contrary, decreased. In addition, SCFA production could serve as a potential biomarker of pediatric obesity, reflecting gut microbiota metabolic activity. Current evidence suggests that children with obesity often exhibit higher fecal SCFA levels; however, further studies are needed to confirm these associations and clarify their underlying mechanisms. Diets play a key role for shaping and modulating the microbiota in children. MD has been shown to enhance bacterial diversity and decrease inflammation, whereas WD, which are rich in sugar, fat and salt, have been shown to decrease microbiota diversity and increase inflammation. Vegetarian diets are beneficial for promoting the production of SCFA. However, the differences found in the findings in children may be due to several factors as age, growth, gender or genetics. It is needed to perform larger studies in pediatric individuals due to the significant impact of different diets on children’s microbiota and thus, in overall health status.

## Data Availability

No datasets were generated or analysed during the current study.
